# An integrated framework to guide evidence-informed public health policymaking

**DOI:** 10.1057/s41271-024-00535-9

**Published:** 2025-01-11

**Authors:** Michelle M. Haby, Ludovic Reveiz, Rebekah Thomas, Helen Jordan

**Affiliations:** 1 Department of Chemical and Biological Sciences, Faculty of Biological and Health Sciences, Universidad de Sonora, Hermosillo, Mexico; 2 Melbourne School of Population and Global Health, The University of Melbourne, Parkville, Australia; 3 Evidence and Intelligence for Action in Health, Pan American Health Organization, Washington, DC USA; 4 Science Division, World Health Organization, Geneva, Switzerland

**Keywords:** Public health policy, Evidence-informed policymaking, Deliberative process, Conflict of interest, Stakeholder

## Abstract

Evidence-informed policymaking emphasizes that policy decisions should be informed by the best available evidence from research and follow a systematic and transparent approach. For public health policymaking we can learn from existing practices of transparent, evidence-informed decision-making for clinical practice, medicines, and medical technology. We review existing evidence-to-decision frameworks, as well as frameworks and theories for policymaking to address the political dimension of policymaking, and use this analysis to propose an integrated framework to guide evidence-informed policymaking. The framework includes nine decision-making criteria and allows for the addition of other context-specific criteria. It also emphasizes elements of the decision-making process that can give greater legitimacy, fairness, and transparency to the policy decision, such as the use of deliberative processes and assessment of conflicts of interest. We offer the framework as a tool to help government policy makers use evidence in a structured and transparent way when making decisions about public health policy options.

## Key messages

- The COVID-19 pandemic highlighted the need for public health decision-making that is informed by evidence from research, transparent, and free of conflicts of interest.
- The integrated framework described here builds on existing and widely used evidence-to-decision frameworks with more explicit consideration of the political factors that influence policymaking.
- The integrated framework emphasizes the need for clear criteria for decision-making along with a transparent, deliberative process that includes all relevant stakeholder groups without conflicts of interest.

## Introduction

Evidence-informed policymaking emphasizes that policy decisions should be informed by the best available evidence on effectiveness, equity, feasibility of implementation, affordability, sustainability, and acceptability to stakeholders within the specific context that the policy is to be applied [1, 2]. It is widely accepted that policies informed by research evidence will be more effective than those that are not, make more effective use of scarce resources, have more legitimacy, and ensure transparency and accountability in decision-making [1–3]. Ideally, evidence-informed policymaking follows a systematic and transparent approach across all aspects of the decision-making process. In reality this is not always the case, and perhaps it is too much of an ‘ask’ to expect that it be so at all times. Policy that is developed under situations where fast decisions need to be made, where there is a perception of low quality or scarce evidence, or when conflicts of interest are involved, can ‘test’ and work against the concept of evidence-informed policy. However, better use of research evidence and transparency may not need to be sacrificed if a framework to guide policy makers under ‘realistic’ policymaking conditions is used.

During the COVID-19 pandemic considerable attention was given to the importance of transparent decision-making that is informed by evidence. Much was written about the decisions that were made and the consideration (or not) of research evidence [4–6]. The WHO and many national governments have conducted reviews of what went well and what could have been done better [7, 8]. Recommendations include calls for greater transparency in decision-making to gain public trust, especially when there is limited research evidence, to publicly release the modeling and evidence used in government decision-making [8], and the use of evidence-based strategies [7] at both national and global levels. This has had implications for governments. In some instances, governments have successfully had legal proceedings brought against them for not acting on the evidence and taking timely action to prevent deaths [9]. Alongside this there are international calls for action to make use of the increased focus on evidence to strengthen the evidence-informed policymaking system [2, 10, 11]. According to the Global Commission on Evidence to Address Societal Challenges, “*COVID-19 has created a once-in-a-generation focus on evidence among governments, businesses and non-governmental organizations, many types of professionals, and citizens*” [10].

We can learn from existing practices of transparent, evidence-informed decision-making in health. These include systems for the development and implementation of guidelines [12–14], health technology assessments [15, 16], and essential medicines lists [17, 18] to guide health care and public health policy. These processes combine clear criteria for decision-making with a transparent, deliberative process for making recommendations and/or decisions [19]. Similar processes and criteria can also be used for coverage decisions, priority-setting, and health benefit package design, though there is not a widely used set of criteria or established process for these [20–24]. Thus, they are not explicitly included in this analysis. In the case of health policymaking outside of clinical practice and medical technology, however, more work needs to be done to institutionalize the use of evidence in a transparent, deliberative process. This is exemplified in WHO’s Evidence-Informed Policy Network Call to Action to accelerate the institutionalization of evidence-informed decision-making, including through the use of high-quality norms, standards and tools [25, 26], and related work to make the use of evidence in policymaking routine [27].

Guidance for evidence-informed policymaking that is systematic and fosters transparency need to factor in the political processes (institutions) and external stakeholders (interests) that play a role in the success or failure of a policy intervention, even when supported by high-quality research evidence [28–31]. Powerful interest groups can and do influence policy by implementing a range of strategies to obfuscate or undermine the evidence used to inform policy, see for example Gómez 2019 [32] and Gilmore et al. 2023 [33]. To respond to, and counteract this, Reich advocates for the use of political economy analysis, alongside the technical analysis, to assess the political landscape, including mapping the key stakeholders and estimating the feasibility of policy change [34]. He argues that this analysis can help the health sector to improve the effectiveness of its policy process, and to give them ideas and strategies on how to shape health policies and the policy process [34, 35]. Likewise, Walt and colleagues argue for the use of health policy analysis to understand the actors, processes and context in which policy is made, in addition to the policy content [36]. They suggest that health policy analysis can be used prospectively (as well as retrospectively) to understand and influence policy outcomes [36–38]. Gómez, another key scholar in this field, notes that politics is an indispensable part of global health policy discussions; and that the just allocation of health resources requires democratic deliberation [39].

In this paper we propose an integrated framework to guide evidence-informed policymaking that builds on existing evidence-to-decision frameworks [14, 15, 18, 19, 40], but includes a more explicit consideration of the political factors that influence policymaking [28–31]. It does so by integrating the political and deliberative process factors into existing, widely used evidence-to-decision frameworks, rather than treating the political and technical factors separately.

### Frameworks for evidence-informed decision-making

**Fig. 1:**
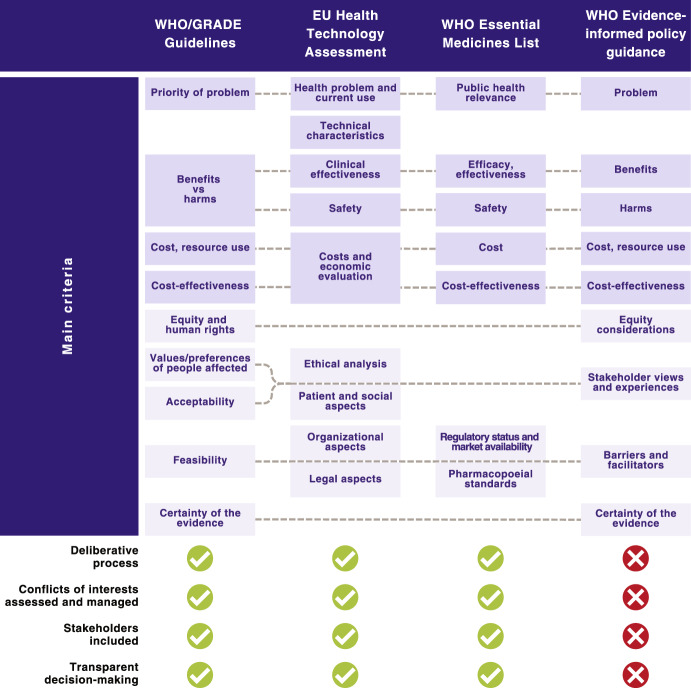
Evidence-informed decision-making frameworks and processes. The guiding manuals and articles for each of the frameworks are: *WHO/GRADE Guidelines* the WHO Guideline Handbook and GRADE articles [13, 14, 19, 40]; *EU Health Technology Assessment* The HTA Core Model® [15, 50, 51]; WHO Essential Medicines List—WHO regulations and manuals [17, 18, 54]; and *WHO Evidence-informed policy guidance* WHO evidence briefs for policy guiding manual [53]. *EU* European Union; *GRADE* Grading of Recommendations Assessment, Development and Evaluation; *WHO* World Health Organization

A selection of existing frameworks that support evidence-informed decision-making for the development of guidelines, health technology assessment (HTA), and the compilation of essential medicine lists (EML) are shown in Fig. 1. This selection was informed by reviews of existing evidence-to-decision frameworks [41–43], an international survey of decision-makers [44], and literature on the use of deliberative processes for decision-making [16, 20, 45–48]. We selected frameworks that are endorsed by the World Health Organization for use by Member Countries [13, 14, 17, 18] due to their likely widespread global use, including in low- and middle-income countries. In the case of HTA, the WHO does not endorse a specific framework or decision criteria apart from cost-effectiveness, though it does offer guidance on the institutionalization of HTA mechanisms [49]. Thus, for HTA we have included the HTA Core Model that has been adopted by the European Union for joint assessments by its Member States [15, 50, 51] due to its wide coverage. In the case of evidence-informed policymaking, there is not a widely used or endorsed framework or process for decision-making. However, the WHO does offer guidance for preparing evidence briefs for policy and for organizing policy dialogues [52, 53], which are aimed at informing the policymaking process—these are included in Fig. 1. The figure includes the main criteria recommended for decision-making, and also indicates some key elements of the decision-making process that would promote a more balanced, non-biased assessment of the evidence and decision-making criteria. These elements include whether a deliberative process is used for decision-making, there is a clear process for assessment and management of potential conflicts of interest, stakeholders are included, and whether there is transparency in the decision-making process. Here, transparency refers to the publishing of the criteria used, stakeholders involved, and outcome of the decision-making process.

The criteria for decision-making that are common between all four frameworks include the priority of the health problem (magnitude or burden, causes), the balance of health benefits (efficacy, effectiveness) and harms/safety, cost/resource use, and cost-effectiveness (Fig. 1). Other criteria that are used in two or more of the four processes include equity considerations, certainty of the evidence, acceptability to stakeholders, and feasibility. In the case of acceptability, the key stakeholders can include the public (patients or population affected), government, deliverers of the intervention, industry, and civil society organizations, depending on the policy option or intervention being assessed. The acceptability of an option overlaps with the values/preferences of people affected, ethical analysis, patient and social aspects, and stakeholder views and experiences, as shown by the dashed lines in the figure. Further, different stakeholders may implicitly consider criteria, such as equity and feasibility, when deciding whether an option is acceptable to them. Feasibility overlaps with resource considerations, and organizational aspects, e.g., existing infrastructures, legal aspects, regulatory status and market availability and availability of pharmacopoeial standards in the case of medicines, among others [14, 15, 18, 51]. All four frameworks and processes shown in Fig. 1 use systematic reviews of primary studies as an important source of research evidence to address the main criteria.

The use of deliberative processes, involving key stakeholders, to consider and weigh the different criteria when making recommendations or decisions about options is an essential part of the WHO/GRADE guidelines development process [13, 14]. Deliberative processes are also used in WHO EML decisions [18]and for HTA [49, 50]. Here, deliberation refers to a discussion that involves the careful and serious weighing of reasons for and against the options [55], and allows “individuals with different backgrounds, interests and values to listen, understand, potentially persuade and ultimately come to more reasoned, informed and public-spirited decisions” [56]. The use of deliberative processes gives legitimacy to the decisions made and their acceptability to stakeholders, especially if they are seen as fair, and are also transparent, allowing accountability [20, 57]. In the case of WHO/GRADE guidelines, EU HTAs, and WHO EML, it is normal practice to publish details of the process, the stakeholders involved, the evidence used, and the criteria assessed [14, 18, 50, 58, 59], though the implementation of this within countries may vary. In the case of the WHO EML, however, some authors have noted that there may be room for improvement in the quality and transparency of the process and for selection of stakeholders [60–62]. In the case of HTA, transparency in decision-making is also recommended by the WHO for countries working towards institutionalization of HTA mechanisms [49], though the implementation of this can vary between countries [16, 47]. The assessment and management of both financial and nonfinancial conflicts of interest is also essential to any evidence-informed decision-making process to ensure credible, unbiased decision-making [11, 63]. It is required when developing WHO/GRADE guidelines [13, 14], for EU HTAs [50], and for the WHO EML process [54]. It is also recommended by WHO for health technology assessments [49], though is not necessarily done at a country level.

In contrast to the process for guidelines, HTA and EML, the process for evidence-informed policymaking does not routinely include a transparent deliberative process, where the process for involvement of stakeholder groups or management of conflicts of interest is outlined. While the WHO evidence-informed policy guidance includes the need to present evidence on many of the key criteria listed in Fig. 1, (e.g. priority of the problem, harms/safety, cost/resource use, and cost-effectiveness) there is no guidance for how policy decisions should be made [53].

### Frameworks/theories for policymaking

The reality of policymaking, including public health policy, is complex. While the policy cycle with its sequentially presented steps of agenda setting, policy formulation/design, decision-making, implementation, and evaluation has been proposed as an ‘ideal’ set of ordered steps [64, 65], the reality is often very different. In practice, the process rarely follows these steps [66]. To better understand how policy is made, a range of theories and frameworks have been proposed. These include Kingdon’s Multiple Streams Theory [31, 67], the Advocacy Coalition Framework [68], the “3-i” framework [69–71], the Narrative Policy Framework [72], and the problem driven political economy framework [73, 74]. A summary of these theories/frameworks is presented in Table 1.

**Table 1:** Policy analysis theories/frameworks

Model and reference	Description	Factors affecting policy decisions	Comments/examples
Kingdon Multiple Streams Theory [31, 67]	Three streams flow through the policy system: **problems**, **policies** (solutions), and **politics**. At critical points in time “**policy windows**” are opened, creating the opportunity for **policy entrepreneurs** to link the three streams together, making policy change more likely to occur	Problem—indicator, focusing event, feedback Policy (solution)—technical feasibility, values, resource adequacy, acceptability to public and politicians Politics—national mood, party ideology, interest groups / balance of interests, Window of opportunity Policy entrepreneur	Windows are more likely to open due to change in the political or problem streams, than in the policy stream. “A worked out, viable proposal, available in the policy stream, enhances the odds that a problem will rise on a decision agenda” page 195 [31]. “Without the presence of an entrepreneur, the linking of the streams may not take place” page 182 [31]
“3-i” framework [69–71]	This framework holds that policy developments and choices are influenced by actors’ **interests** and **ideas**, as well as by **institutions**	Ideas—people’s beliefs (includes research evidence), people’s values (includes cultural norms) Interests—interest groups/stakeholders (including researchers) Institutions—government structures, policy networks, policy legacies, characteristics of the policymaking process	Some authors also refer to the importance of networks interacting with the 3-i’s [29], or to external factors that fall outside of the policy choice being analyzed (e.g. an election) [85]
Narrative Policy Framework [72]	This framework recognizes the power of **stories** used by policy actors in seeking to influence decision-making. Stories contain the elements of **setting**, **characters**, **plot** and **moral**	The narrative or story: Setting—context, including institutional and socio-economic factors Characters—contains at least one actor, e.g., a hero or a villain Plot—provides the arc of action, e.g., overcoming adversity, villains causing trouble, suffering of victims Moral—describes the cause of, and solution to, the policy problem [86]	“This framework can be viewed as a separate policy framework, or as embedded within other frameworks, such as multiple streams theory, where narratives can influence one or more streams, and be harnessed by policy entrepreneurs” [75] “This suggests that research evidence may be more persuasive when translated or integrated into narrative elements such as setting, moral of the story, characters and plot” [75]
Problem driven political economy framework [73, 74]	This framework starts with a problem or issue for which a **technical and economic analysis** of feasible solutions has not worked. It then adds a political economy analysis focused on: a) relevant **structural factors**, b) existing **institutions** (formal and informal), and c) **stakeholder interests**, constellations, and power	Technical—effectiveness, cost-effectiveness etc Political: Structural factors—country demographics, geography, socio-cultural factors, etc Institutions—the ‘rules of the game’, local laws, conventions, traditions Stakeholders—individuals, organizations, coalitions from the public, private or civil society sectors	“[Development] outcomes are achievable when we consider the intersection of politically possible and technically sound” [74]. The framework has been used to identify and explain barriers and enablers to implementing nutrition and sustainability policy into government food procurement [87], and in an analysis of sugar-sweetened beverage taxation in three Latin American countries [88], among others
Advocacy Coalition Framework [68]	This framework considers the policy subsystem as the primary unit of analysis, and includes the “policy scope, territorial scope and actors directly or indirectly influencing policy subsystem affairs” (p189)	Relative stable parameters (e.g. social, physical and, constitutional/ institutional structures) Dynamic external events, e.g., socioeconomic conditions, public opinion, composition of coalitions	

These policy theories/frameworks highlight important elements of influence that are not sufficiently considered in the evidence-to-decision frameworks shown in Fig. 1 and includes the role of different *interest groups and stakeholders* and coalitions of, in influencing policy change. In Kingdon’s multiple streams theory, the influence of interest groups is included in the politics stream, as well as in the role of the policy entrepreneur, that is, a person typically within government working alone or in conjunction with a network or coalition of supporters, to identify and/or facilitate the synchronous ‘running’ of the three streams or opening of the policy window [31]. The role of different stakeholders in influencing policy change is also evident in the Advocacy Coalition Framework, 3-i and problem driven political economy frameworks [68–71, 73, 74], while stakeholders can use narratives to influence policy as underpinned by the Narrative Policy Framework [72, 75].

Another gap in the evidence-to-decision frameworks is recognition of the role that *institutions and structural factors* present when making policy decisions. These include government structures, policy legacies, and characteristics of the policymaking process. These factors are considered to various degrees within the 3-i, problem driven political economy, and Advocacy Coalition Frameworks [68–71, 73, 74] and form the setting for the narrative policy framework [72]. While these frameworks are predominantly used to explain how policy is made, they emphasize the importance of the political, institutional and structural factors contributing to and influencing policy. Any guidance in evidence-informed policymaking needs to consider these factors if deliberative decision-making processes that are transparent and ‘power’ neutral, are to be institutionalized.

### An integrated framework for evidence-informed policymaking

**Fig. 2:**
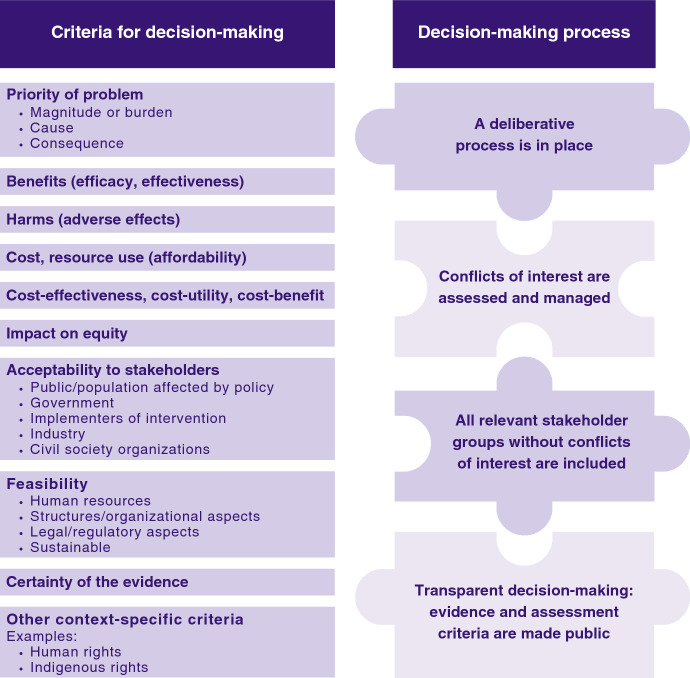
An integrated framework to guide evidence-informed policymaking

The frameworks/theories of policymaking presented in the previous section help to explain the reality of policymaking, and the many influences, that limit or over-ride the balanced use of ‘evidence’. In this section we propose an integrated framework for evidence-informed policymaking (Fig. 2). The proposed integrated framework shown in Fig. 2 incorporates the five criteria for decision-making that are common across the four evidence-to-decision frameworks presented in Fig. 1 [13–15, 17, 18, 51]. These include the priority of the problem, benefits, harms, cost and resource use, and cost-effectiveness. Other criteria added to the proposed framework (Fig. 2) include impact on equity, acceptability to stakeholders, feasibility, and certainty of the evidence; these are used in two or more of the four frameworks from Fig. 1. Ethics has not been included as a specific criterion but is reflected in the equity and acceptability criteria and in the decision-making process elements that ensures procedural fairness [23].

In contrast to existing evidence-to-decision frameworks, we have included additional sub-criteria for ‘acceptability’ which separate out the different types of groups for whom policy ‘acceptability’ is gauged. This is informed by our review of frameworks/theories for policymaking (Table 1) that highlight the importance of different interest groups (and actors), including those with vested interests, in influencing policy change. This deliberate separation of the different stakeholder groups aims to facilitate a more transparent consideration of all stakeholder viewpoints, values and preferences in the decision-making process and to prevent or make more transparent any viewpoints or ‘pressures’ exerted from those with vested interests. We have also included sub-criteria for the ‘feasibility’ criterion to highlight the importance of evidence on the applicability, or implementability, of the proposed policy in the local context. This also serves to highlight factors that can affect the transferability of global evidence to the local context [76, 77]. In addition to the previously mentioned criteria, the framework allows for the addition of criteria that may be relevant to the specific context or values of those that may be affected by the policy, such as human rights, indigenous rights, and environmental impact.

An important contribution of the proposed integrated framework is the emphasis on elements of the decision-making process that, when implemented can give greater legitimacy, fairness, and transparency to the policy decision. These also serve to emphasize the frequent limitations in current policymaking, such as lack of transparency and undue influence of industry stakeholders that have clear conflicts of interest [33]. For our proposed strengthened framework, we have explicitly called for the exclusion of stakeholders with conflicts of interest from the decision-making process. While these decision-making process elements are not routinely implemented in all decision-making contexts shown in Fig. 1, they do serve to highlight how decisions *should* be made to ensure fairness and accountability [2, 20, 46–48].

While previous work has also shown similarities in the decision criteria (and sources of evidence) between the different evidence-informed decision-making frameworks [2, 11, 78, 79], this paper takes the work further by examining the decision-making process in more detail—including the use of deliberative processes, the assessment and management of conflicts of interest, the inclusion of relevant stakeholder groups without conflicts of interest, and whether the process was transparent. It goes beyond previous work in giving more attention to the case of evidence-informed decision-making for public health policy, rather than the previous focus on medicines, technology, and healthcare [78, 79].

### Applications of the proposed integrated framework to guide evidence-informed policymaking

The principal application of the proposed framework is to help government policymakers use evidence in a structured and transparent way to inform their decisions about public health policy options. This is aided by the decision-making criteria on the left side of Fig. 2 for which the best available evidence for each criterion should be presented. Further, separating out the different stakeholder groups within the ‘acceptability to stakeholders’ decision-making criteria makes the potential influence of the different interest groups more transparent to the public. Further, by excluding stakeholders with conflicts of interest from the decision-making process and ensuring that the process is transparent, the influence of vested interests is reduced. This process should enable policymakers to defend their decisions, even when evidence is limited.

A secondary application of our proposed framework is for comparative policy analysis. For this it is helpful to consider questions (Table 2) that serve to highlight factors that can influence the acceptability of a policy option. These include the ideology of the elected government and the national mood. The proposed framework and questions for reflection can be used for retrospective analyses to identify factors that contributed to success or failure of a policy proposal. It can also be used prospectively to analyze and strengthen a policy proposal and approach to increase its chance of success [80], e.g., by framing the problem in a way that will motivate different groups [81], or by designing the policy to make it more acceptable to key stakeholders or more feasible to implement.

**Table 2:** Questions for reflection to assess and strengthen the evidence-informed policymaking process

Questions
1. Is the problem a government priority? To what degree is the issue recognized, understood, and currently being acted on?
2. How is the problem framed? Does this framing make it more acceptable to government policymakers?
3. Has there been a focusing event or change in an indicator that increases the importance of the problem?
4. Could the design of the policy option be modified (or framed) to make it more acceptable to government policymakers?
5. Is there a policy champion/entrepreneur involved that supports the proposal?
6. Is there a strong, united coalition of supporters for the proposed policy option?
7. Does the proposed policy option (solution) align with party ideology?
8. Does the proposed policy option (solution) align with the national mood?

Finally, the framework can be used to advocate for the institutionalization of more transparent, evidence-informed policymaking, which is the only way to achieve lasting change in the policymaking process [30]; particularly if there is a strong mandate, such as through legislation, incorporation in government structures, and commitment of resources. The framework serves as a roadmap for what needs to be considered in evidence-informed policymaking. An example of where this has been achieved is the formalization of the Department of Health Technology Assessment and Evidence-Based Health in the Chilean Ministry of Health in 2017 to support decision-makers, including for public health policies [82]. Another example in the Americas Region is Brazil with the embedding of the Evidence-Informed Policy Network in the Brazilian Government Ministry of Health [83, 84].

We offer the framework as a tool to help government policy makers, and those who support them, use evidence in a structured and transparent way when making decisions about public health policy options. We encourage others to test whether this integrated framework is useful for public health policy decision-making, leads to policies that are effective and fair, and improves transparency and accountability in government decision-making.

## Conclusion

This paper presents an integrated framework to support evidence-informed policymaking. It builds on existing and widely used evidence-to-decision frameworks with more explicit consideration of the political factors that influence policymaking. These include the influence of different interest groups/stakeholders, including those with clear conflicts of interest, and the importance of political, institutional and structural factors in influencing policy change. The integrated framework includes the criteria: priority of the problem, benefits, harms, cost and resource use, cost-effectiveness, impact on equity, acceptability to stakeholders (with sub-criteria to differentiate the key stakeholder groups), feasibility (with sub-criteria for the different factors that can affect applicability and implementation in the local context), and certainty of the evidence. It also allows for the addition of other context-specific criteria, such as human rights and environmental impact. The proposed framework also emphasizes elements of the decision-making process that, when implemented can give greater legitimacy, fairness and transparency to the policy decision. These include the use of deliberative processes, the assessment and management of conflicts of interest, inclusion of relevant stakeholder groups without conflicts of interest (and exclusion of stakeholders with conflicts of interest from the decision-making), and transparency in the decision-making process.

## Data Availability

Not applicable.
